# Career Crafting Training Intervention for Physicians: Protocol for a Randomized Controlled Trial

**DOI:** 10.2196/18432

**Published:** 2020-10-08

**Authors:** Evelien H van Leeuwen, Machteld van den Heuvel, Eva Knies, Toon W Taris

**Affiliations:** 1 University Medical Center Utrecht Utrecht Netherlands; 2 Department of Work and Organizational Psychology University of Amsterdam Amsterdam Netherlands; 3 Utrecht University School of Governance Utrecht University Utrecht Netherlands; 4 Department of Social, Health and Organizational Psychology Utrecht University Utrecht Netherlands

**Keywords:** career crafting, job crafting, career self-management, intervention study, employability, physicians, intervention mapping

## Abstract

**Background:**

Physicians work in a highly demanding work setting where ongoing changes affect their work and challenge their employability (ie, their ability and willingness to continue working). In this high-pressure environment, physicians could benefit from proactively managing or *crafting* their careers; however, they tend not to show this behavior. The new concept of career crafting concerns proactively making choices and adapting behavior regarding both short-term job design (ie, job crafting) as well as longer-term career development (ie, career self-management). However, so far, no intervention studies have aimed at enhancing career crafting behavior among physicians. Given that proactive work and career behavior have been shown to be related to favorable outcomes, we designed an intervention to support career crafting behavior and employability of physicians.

**Objective:**

The objectives of this study were to describe (1) the development and (2) the design of the evaluation of a randomized controlled career crafting intervention to increase job crafting, career self-management, and employability.

**Methods:**

A randomized controlled intervention study was designed for 141 physicians in two Dutch hospitals. The study was designed and will be evaluated based on parts of the intervention mapping protocol. First, needs of physicians were assessed through 40 interviews held with physicians and managers. This pointed to a need to support physicians in becoming more proactive regarding their careers as well as in building awareness of proactive behaviors in order to craft their current work situation. Based on this, a training program was developed in line with their needs. A number of theoretical methods and practical applications were selected as the building blocks of the training. Next, participants were randomly assigned to either the waitlist-control group (ie, received no training) or the intervention group. The intervention group participated in a 4-hour training session and worked on four self-set goals. Then, a coaching conversation took place over the phone. Digital questionnaires distributed before and 8 weeks after the intervention assessed changes in job crafting, career self-management, employability, and changes in the following additional variables: job satisfaction, career satisfaction, work-home interference, work ability, and performance. In addition, a process evaluation was conducted to examine factors that may have promoted or hindered the effectiveness of the intervention.

**Results:**

Data collection was completed in March 2020. Evaluation of outcomes and the research process started in April 2020. Study results were submitted for publication in September 2020.

**Conclusions:**

This study protocol gives insight into the systematic development and design of a career crafting training intervention that is aimed to enhance job crafting, career self-management, and employability. This study will provide valuable information to physicians, managers, policy makers, and other researchers that aim to enhance career crafting.

**International Registered Report Identifier (IRRID):**

RR1-10.2196/18432

## Introduction

Physicians work in a highly demanding work setting where ongoing changes affect their work. Physicians’ work environment is characterized by high work pressure and other stressors [1]. This challenges physicians’ ability and willingness to continue to work until the retirement age in their current profession [2] (ie, their employability [3]). Recent studies show that the employability profile of physicians and other workers in the health care sector is relatively low compared to employees in other sectors. Specifically, in a study among 42,613 health care workers in the Netherlands, 47% of them thought it was possible to find employment beyond their current employer, compared to 57% of employees in other sectors; 52% of health care workers regularly perceive a high physical workload, compared to 38% of employees in other sectors; and 19% of health care workers often perceive a high emotional workload, compared to 7% of employees in other sectors [4].

In order for employees to successfully navigate within this complex environment, they must proactively take control over their working life by creating a resourceful, healthy, and motivating environment for themselves [5]. This can be done through career crafting, which is a relatively new concept in the literature, and is defined as “individuals’ proactive behaviors aimed at optimizing career outcomes through improving person-career fit” [6]. Career crafting entails both choices and changes to the current work environment (ie, job crafting) as well as actions focused on longer-term career design (ie, career self-management) [6]. Job crafting refers to the self-initiated behaviors that employees take to shape, mold, and change their jobs [7-9]. For example, people can craft social resources such as support or they can optimize tasks or situations that are hindering. An example of job crafting is limiting tasks that drain energy, such as reducing the time spent on meetings by 15 minutes per meeting. Career self-management is defined as the extent to which employees proactively develop their careers as expressed by diverse career behaviors [10]. An example of career self-management is networking behavior, in which someone proactively approaches others who can be helpful in shaping their career. Career crafting entails the combination of both types of behaviors. For example, an employee may reduce energy-draining activities (ie, through job crafting) by communicating firmer boundaries in meetings (eg, “I have 30 minutes for this meeting; what are our highest priorities?”). The time thus gained is used to proactively network with someone from another organization (ie, career self-management), who is employed in a position that is of interest to the employee, to learn about how he or she managed to get that position.

Career crafting is considered an important individual behavior aimed at safeguarding the sustainability of one’s career over time [6]. This suggests that career crafting may possibly enhance employability. However, empirical evidence about the antecedents and consequences of career crafting is lacking and requires further examination. Previous studies have found that career crafting behaviors such as job crafting and career self-management fulfil important roles in contemporary careers and result in beneficial outcomes [11,12]. Previous studies found that career crafting behaviors are beneficial to the individual, as reflected in enhanced work engagement [13], well-being [14], meaningfulness, and job satisfaction [15], as well as to the organization, as reflected in enhanced performance [16]. This makes it worthwhile to invest in an intervention program that enhances physicians’ career crafting and employability, which is urgent in today’s health care career context.

Three gaps exist in the current literature. First, despite the increasing importance of proactive career behaviors, to our knowledge, as yet no intervention studies have aimed at enhancing career crafting. Career adaptability training for graduates [17] focused on facilitating the school-to-work transition but did not examine how to stay employable within a work context. That study and other existing career interventions had a different focus (eg, career coaching or counseling) than the subject of our study or showed methodological weaknesses [18]. These studies, for instance, used a cross-sectional study design [19], lacked a control group [20-22], or did not assign participants randomly to a control or treatment group, as shown in a meta-analysis by Whiston et al [23].

Second, career studies have mainly focused on employees in general [24], while employees in different jobs have been shown to have different career trajectories and employment opportunities [25]. Paying attention to physicians’ careers is important for two reasons. First, some studies describe their career choices as serendipitous or circumstantial [26] and mention that physicians are neither actively engaged in career planning nor being stimulated to do so [27,28]. Other studies have shown that attention on careers is beneficial for employee job satisfaction [22] and may help employees to keep up with a fast-changing work environment [29]. Second, physicians’ career trajectories are different from those of other employees. Physicians usually finish their medical training around the age of 30 years and work as medical specialists for the next 30-35 years of their career. Although their high level of education may stimulate career possibilities, the specialized nature of their work tends to reduce their employment opportunities and may, thus, result in physicians often having the same job until retirement [25]. Relevant career opportunities for physicians should, therefore, not only focus on promotion, since possibilities for this are limited, or on external opportunities (eg, changing jobs or organizations), but they should also include possibilities of internal career opportunities (eg, changing work content or tasks). Focusing on physicians’ career content may help physicians in developing coping skills to effectively deal with the challenges presented by their work environment. This seems important as research has shown that some physicians are insecure about their competencies to fulfil nonclinical tasks, such as teaching, managerial skills, and financial skills, for which they are not primarily educated [30,31]. The career crafting training developed in this study is likely to fit physicians’ needs, since their needs have been identified and because the content of the training has been developed in collaboration with physicians.

Third, most intervention studies mainly focus on the analysis of outcomes and lack a systematic process evaluation [32,33]. This may be partly explained by the absence of an evidence-based framework that describes the elements that need to be included in process evaluations. Nevertheless, process evaluation is important as it helps us to understand why parts of an intervention result in a certain outcome and shows how research findings can be used to guide practice [32].

In responding to these knowledge gaps, this study makes the following contributions. This study contributes to the literature on proactive career behavior by elaborating on the development and design of the evaluation of a career crafting training intervention. In doing so, the specific needs and challenges that physicians face are taken into account, which increases the practical utility of this intervention. This paper elaborates on the systematic process in which this career intervention is developed for, and in collaboration with, physicians. Furthermore, the research protocol discusses a framework to conduct a process evaluation, based on the current literature. The objectives of this study are to describe (1) the development and (2) the design of the evaluation of a randomized controlled career crafting intervention developed for physicians to increase job crafting, career self-management, and employability.

## Methods

### Overview

The intervention was developed in a systematic way, using elements of the intervention mapping (IM) protocol. IM is a widely accepted methodology for planning theory-based and evidence-based health promotion programs [34] and has been used in numerous studies, eg [35], [36]. IM consists of six steps: (1) needs assessment, (2) definition of program objectives, (3) methods and practical applications, (4) intervention program development and pilot test, (5) adoption and implementation, and (6) evaluation. The completion of every step creates a product that is the guide for the subsequent step [34]. Although these steps suggest that this is a linear process, moving back and forth between the steps is part of the process.

### Step 1: Needs Assessment

The first step of IM was to assess and understand the problem and needs of the participants [34]. This intervention was custom-made in close collaboration with potential participants, physicians, and other stakeholders, such as managers of physicians who also work as physicians. There is widespread agreement that a participative approach in the design of interventions is promising. Employees are often familiar with the problems and the best solutions in their work context, and people can identify better with a project if they perceive themselves to be the agents of change rather than the objects of change [37].

In an earlier stage of this study, 40 face-to-face exploratory interviews were conducted to examine physicians’ experiences with job crafting, career self-management behavior, and employability. Out of the 40 interviews, 27 (68%) were done with the target population, namely physicians, and 13 (33%) were conducted with their managers who also worked as physicians. The results of these interviews were discussed and interpreted by a planning group. This group consisted of the researchers of this study, a senior board member and a physician who also worked as a manager in the academic hospital, and two physicians and a senior board member of the general hospital. The reflections of the planning group were also discussed in both hospitals with people from the human resources department who were familiar with current policies and trainings for physicians. The interviews revealed that physicians lack attention for job crafting and career self-management. Moreover, employability was hardly discussed or thought of in this occupational group. Despite this, physicians and their managers emphasized the importance of finding ways to increase physicians’ employability. They described several challenges: dealing with a high workload, rapid technological developments, finding a healthy work-life balance, the need to fulfil nonmedical-related tasks (eg, educational tasks or being part of certain committees), and the repetitive character of their tasks, which challenged their motivation in the longer run. Both physicians and managers mentioned that these challenges affected their ability and/or willingness to continue to work in their profession. Some physicians also indicated that support in these areas would be helpful, since a focus on these themes was not part of their standard medical training. Career crafting training, which helps them to cope with their current work environment and prepare them for their further career, is therefore expected to be in line with their needs.

### Step 2: Definition of Program Objectives

The next step involved specifying the change objectives. This included what must be changed and who must make the change [34]. These were formulated based on the needs that physicians and managers expressed in step 1. The following three program objectives were chosen: the intervention will increase physicians’ (1) job crafting behavior, (2) career self-management behaviors, and (3) employability. Following the IM approach, three personal determinants were identified to realize behavioral change in order to reach these objectives [38]. These were awareness of the importance of investing in job crafting, career self-management, and employability; knowledge about these topics; and learning the skills to know how these investments can be made.

### Step 3: Methods and Practical Applications

In the third step, methods and practical applications were chosen to achieve the objectives [34], based on existing literature and the stakeholder interviews. Multimedia Appendix 1 shows the theoretical methods and the practical applications for each determinant.

### Step 4: Intervention Program Development and Pilot Test

Step 4 included a description of the intervention, completed program materials, and program protocols. The intervention consisted of a 4-hour group training session for diverse groups of physicians with a pre- and postmeasure. This half-day session was a combination of theory, reflection, exercises, and goal setting (see Multimedia Appendix 1). Participants learned the principles of proactive work and career behaviors, and all participants left the session with a plan outlining four small actions for the following 4 weeks.

In order to be successful, the program required pilot testing with intended implementers and recipients [34]. The survey was created and pilot-tested by the first author in 4 face-to-face interviews with physicians and with managers who also worked as physicians. A *think-aloud* method was used, meaning that participants were asked to think out loud when reading the texts and answering the questions. At the end of the interview, some open-ended questions were asked about the survey’s content, wording, and style of addressing physicians. If needed, introduction texts and items were adapted. Then, a list was made including program themes, assignments, and time frame planning. A training program and protocol was drafted, which was pretested in a pilot training session with intended users. A total of 5 physicians participated, who varied as much as possible on variables that might affect the variables of interest (eg, gender and age). The physicians followed the pilot training session and evaluated the training session at the end in a group discussion, based on the following: content, wording, suitability of given examples, and types of exercises. This resulted in optimization of the training content through some adaptations in allocated time and wording to make the content better suited to the perspectives of physicians. Moreover, examples of job crafting and career self-management were added, based on experiences of the physicians.

### Step 5: Implementation

In this step, participants were recruited via presentations in physicians’ staff meetings, word-of-mouth communication in existing networks, and promotions of the training via email. An email with information on the intervention (ie, goal, content, and duration of the intervention) was sent to the heads of departments, who were asked to share the email with physicians on their team. At the same time, the emails were sent to the representatives of physicians, who were asked to send the email to physicians in their department. In addition, accreditation was requested and granted. This means that physicians earned accreditation points (ie, professional development points) when participating in this training, which they need to stay registered. This will likely increase physicians’ extrinsic motivation to participate in this training.

This intervention study started with randomly assigning participants to the waitlist-control group or the intervention group. Two independent randomizations were done using the randomizer function in Microsoft Excel (version 16.41): one for physicians in the academic hospital who were either assigned to the waitlist-control group or intervention group, and one for physicians in the general hospital who were randomized in a waitlist-control group or intervention group. Two independent randomizations enhanced the probability of equally dividing physicians in one hospital to the control or intervention group. This is important given the expectation that physicians from both hospitals differ on characteristics that might affect their career crafting behavior, such as type of contract and the degree of specialization. The advantage of the waitlist-control group is that all physicians received the intervention in the end. They were blind to the condition (ie, waitlist-control group or intervention group) to which they were assigned.

**Figure 1:**
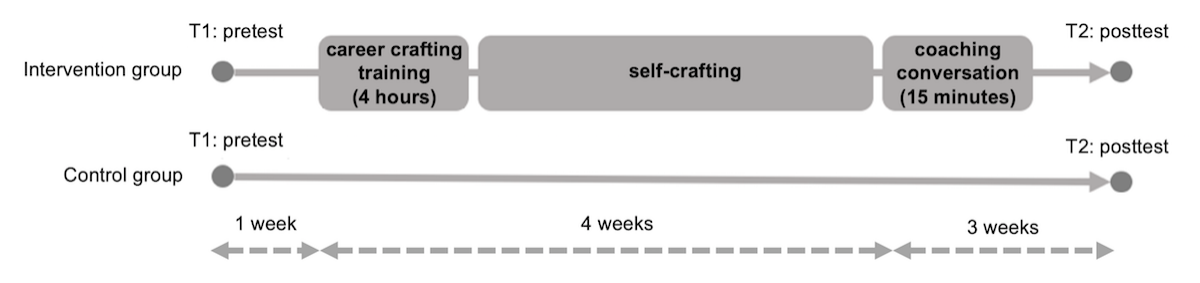
Design of the career crafting intervention.

Figure 1 shows the procedure of this experiment. Participants in both the intervention and control groups received an email inviting them to complete the pretest. A total of 1 week after receiving the digital survey, which was sent through the program Qualtrics (version April 2020), physicians in the intervention group received a 4-hour training intervention. In total, 7-14 physicians were planned to attend each training session. After that, they worked on their self-set goals for the next 4 weeks. Then, a coaching conversation took place on the phone. A total of 3 weeks after the coaching conversation, which was 8 weeks after the pretest, physicians in both groups received a link to the posttest.

### Step 6: Evaluation of the Results

#### Overview

Both the effectiveness as well as the implementation process of the career crafting intervention will be systematically evaluated. The effectiveness of the intervention program will be evaluated quantitatively, by analyzing the variables of interest. The implementation process of the intervention will be examined through a process evaluation, both quantitatively, by examining the answers to survey items, and qualitatively, by asking for experiences of participants after the coaching session on the phone and in an open-ended question at the end of the last survey.

#### Effectiveness and Outcomes

The effectiveness of the career crafting training intervention will be examined by comparing the intervention and control groups on the outcomes that were gathered in the pre- and posttests. The main outcome measures of this study were job crafting, career self-management behavior, and employability. Perceptions on job crafting regarding personal resources were measured (9 items) [39], and perceptions on job crafting to change work aspects were measured (10 items) [7]. Perceptions on career self-management behavior were measured [10] by examining general career behaviors, career planning, career self-exploration, environmental career exploration, networking, voluntary human capital and skill development, and positioning behavior (9 items). Perceived employability was measured by asking for physicians’ willingness and mental and physical ability to continue working in their current profession until the retirement age (3 items) [3]. Additional outcome measures were job satisfaction [40], career satisfaction [41], work-home interference [42], work ability [43], and performance [44]. Additionally, background information was gathered on age, gender, type of employment contract, hours worked according to the contract, organizational tenure, and functional tenure. Data from the pre- and posttests of individuals could be linked with unique codes that were generated by the program Qualtrics.

#### Participants

The sample size was calculated on the basis of 95% power to reject the null hypothesis with a 2-tailed significance level of 5%. Assigning equal numbers of participants to the intervention and control groups, and based on the effects of job crafting training interventions on job crafting behavior [45,46], a total of 120 physicians (60 in each group) were needed. We aimed for 150 participants, to allow for 20% dropout. We widely communicated the possibility of participating in this intervention study to physicians, as explained in step 5. However, we did not reach all physicians (ie, 685 physicians in the academic hospital and 225 physicians in the general hospital), as we were not invited into all the departments of the hospital to give a presentation about the training content. In the end, 141 physicians participated; 107 physicians (76%) were employed by the academic hospital and 34 physicians (24%) worked in the general hospital.

#### Data Analysis

Depending on the assumptions for outliers, normality, and sphericity, we are planning to conduct 2-way, repeated-measures multivariate analyses of variance in SPSS (version 25.0 (IBM Corp)), to assess the *time* × *group* interaction effects of the intervention on the main and additional outcome measures. Subsequently, if the tests for assumptions are not violated, we will perform repeated-measures analyses of variance to further examine the effects within the control and intervention groups.

#### Process Evaluation

A process evaluation will be done during the process of implementing the study, providing insight into factors that may have helped or hindered the effectiveness of the intervention [32]. Despite the lack of an evidence-based framework describing the elements that need to be included in process evaluations [32], three dimensions are often mentioned: (1) context, (2) implementation process, and (3) participant mental models and mechanisms [32,47]. The elements examined within these dimensions are described in Multimedia Appendix 2. Both quantitative and qualitative methods will be used to examine these process evaluation elements, since both methods and the combination of them are shown to be effective [32,37,48].

#### Ethics

The University Medical Center Utrecht confirmed that this study falls outside the scope of the Dutch Medical Scientific Research Involving Human Subjects Act (*Wet medisch-wetenschappelijk onderzoek met mensen* [WMO], in Dutch) and, therefore, formal ethical approval was not required (METc 2019, 19/109). Nevertheless, ethical guidelines were applied as follows: all participants signed a written consent form stating that participation is voluntary, outcomes are held confidentially, and they can withdraw from the study at any time; they were also told that they could change their answers (ie, through a back button) before submitting the survey and were reminded of this at every contact moment. All study material was anonymized and saved on a protected server.

## Results

Data collection was completed in March 2020. Evaluation of outcomes started in April 2020. One researcher conducted the primary analyses; these results were discussed with the research team in July 2020, which resulted in some adjustments and additional analyses. The process evaluation of the qualitative data that were obtained in the coaching interviews was done after the evaluation of the outcomes. Study results were submitted for publication in September 2020.

## Discussion

This article describes (1) the development and (2) the evaluation design of the first career crafting training intervention aimed at increasing job crafting, career self-management behavior, and employability of physicians. This study protocol describes the systematic development of the intervention using parts of the IM protocol.

The strengths of this study boil down to three main points. First, this study addresses a novel concept, career crafting, which refers to proactive work and career behaviors that are linked to positive employee outcomes, such as well-being and employability. An intervention approach seems timely and relevant given the work and career-related challenges that physicians are facing. The intervention can potentially help them to cope with ongoing changes in their work environment [49] and might enhance the sustainability of their careers over time [6]. In order to fit the intervention’s content with physicians’ needs, needs are assessed through 40 interviews. This needs assessment forms the basis of the intervention program, which is further developed in close collaboration with physicians and other relevant stakeholders (eg, managers of physicians). Second, a robust research design is used, namely a randomized controlled field experiment, which is a high-quality approach to examine the causal effects of an intervention [50]. The effect and process evaluation help us to understand the outcomes of the intervention study and can be used to guide practice [32]. A third strength is that we designed the training to take place in 4-hour sessions, which kept the time investment low. The practical applicability of this study, therefore, seems high and the training could possibly be administered in an online format as well. Future studies could use this study protocol to examine such an intervention study in other occupational contexts to gain more insight into the effectiveness of a career crafting training intervention across different contexts with varying cultures.

Apart from the above strengths, this study has some limitations. First, contamination may occur when participants in the intervention group communicate with waitlist-control participants about the content of the training. However, the chances of contamination are small, since physicians are widely spread across the organization. Second, because we use a field experiment, our control is limited. This means that participants might not adhere to the instructions, might be unable to attend the assigned training, might not complete both surveys, or could drop out completely. We deal with these problems by (1) keeping track of participants that want to change groups and (2) sending two reminders by email to complete the survey and four reminders to work on the self-set goals after the training. A third limitation is that in order to minimize dropout, we did not include a long-term follow-up measurement. A second follow-up measurement could have revealed the extent to which findings can be generalized across longer time periods.

In conclusion, the systematic development of the intervention based on parts of the IM protocol has resulted in a 4-hour career crafting group training intervention to support physicians in developing proactive work and career behaviors. Subsequent analyses in a follow-up study can provide valuable insights to physicians, managers, and policy makers about the intervention’s effectiveness for physicians.

## Appendix

Multimedia Appendix 1 Theory-based methods and practical applications of the career crafting intervention.

Multimedia Appendix 2 Elements of the process evaluation.

## Abbreviations

IM: intervention mapping
WMO: Wet medisch-wetenschappelijk onderzoek met mensen (Dutch Medical Scientific Research Involving Human Subjects Act)
