# Supplementary material for: Career Crafting Training Intervention for Physicians: Protocol for a Randomized Controlled Trial
Source: JMIR Res Protoc. 2020 Oct 8;9(10):e18432. doi: 10.2196/18432 (PMC7582143; doi:10.2196/18432)
Supplement: Multimedia Appendix 1 [file resprot_v9i10e18432_app1.pdf]

Determinants of career crafting behaviors	Theoretical method	Practical application
**Awareness of career goals, values and interests**	Self-affirmation	Participants reflect on their achievements, goals, values and what they respect in others in a writing exercise and share this with their peers [38]
	Imagery	Participants do a guided visualization on their ideal work situation in the future [17]
**Knowledge of relevant concepts (job crafting, career self-management, employability)**	Discussion and elaboration	Relevant theoretical concepts are presented and participants are invited to discuss their own understanding of the concepts and what they signify to them [38]
**Knowledge / awareness**	Modelling	a) Throughout the training examples of job crafting and career self-management are shared, where available these examples came from peer-physicians. These examples model possible proactive crafting behaviors to inspire participants<br>b) Participants are invited to reflect and choose a 'career hero', a person they admire or respect for how they have developed their career. In pairs they analyze this person's career choices, proactive behaviors, dealing with obstacles and what their choice means for their own career needs and values [38]
**Skills / proactive behavior efficacy**	Self-monitoring and reflection	Participants reflect on lessons from their own career up till now. In small groups they discuss changes and choices they have made in the past, how they have shown proactive career behavior and how this shaped their career [38]
**Self-efficacy / motivation to act**	Goal-setting, feedback and public commitment	Each participant is invited to formulate self-chosen goals, two actions they intend to take to proactively craft their current work and two actions that contribute to their longer-term career goals. Participants discuss their plans in pairs and each participant shares at least one goal with the group [34,38]
