# Supplementary material for: Career Crafting Training Intervention for Physicians: Protocol for a Randomized Controlled Trial
Source: JMIR Res Protoc. 2020 Oct 8;9(10):e18432. doi: 10.2196/18432 (PMC7582143; doi:10.2196/18432)
Supplement: Multimedia Appendix 2 [file resprot_v9i10e18432_app2.pdf]

Dimension	Elements	Method	Source
**1.Context**	Barriers/facilitators of implementation	One-on-one coaching session	Participants
**2.Implementation process**	Consistency of implementation across intervention groups	Observation	Trainer and researcher
	Fidelity	Compare program plan with observation	Researcher
	Use of intervention in daily practice	Survey, one-on-one coaching session	Participants
**3.Participants mental models**	Readiness for change	Survey	Participants
	Judgment of atmosphere	Survey and in group discussion	Participants, trainer and researcher
	Degree of participation	Survey, observation, one-on-one coaching sessions	Participants, trainer and researcher
	Appreciation of intervention	Survey and in one-on-one coaching session	Participants
	Participants behave as intended, e.g. complied to group assignment	Observation	Researcher
